# Design Features for Improving Mobile Health Intervention User Engagement: Systematic Review and Thematic Analysis

**DOI:** 10.2196/21687

**Published:** 2020-12-09

**Authors:** Yanxia Wei, Pinpin Zheng, Hui Deng, Xihui Wang, Xiaomei Li, Hua Fu

**Affiliations:** 1 School of Public Health Fudan University Shanghai China

**Keywords:** mHealth, design feature, user engagement, thematic synthesis analysis

## Abstract

**Background:**

Well-designed mobile health (mHealth) interventions support a positive user experience; however, a high rate of disengagement has been reported as a common concern regarding mHealth interventions. To address this issue, it is necessary to summarize the design features that improve user engagement based on research over the past 10 years, during which time the popularity of mHealth interventions has rapidly increased due to the use of smartphones.

**Objective:**

The aim of this review was to answer the question “Which design features improve user engagement with mHealth interventions?” by summarizing published literature with the purpose of guiding the design of future mHealth interventions.

**Methods:**

This review followed the PRISMA (Preferred Reporting Items for Systematic Reviews and Meta-Analyses) checklist. Databases, namely, PubMed, Web of Science, Cochrane Library, Ovid EMBASE, and Ovid PsycINFO, were searched for English and Chinese language papers published from January 2009 to June 2019. Thematic analysis was undertaken to assess the design features in eligible studies. The Mixed Methods Appraisal Tool was used to assess study quality.

**Results:**

A total of 35 articles were included. The investigated mHealth interventions were mainly used in unhealthy lifestyle (n=17) and chronic disease (n=10) prevention programs. Mobile phone apps (n=24) were the most common delivery method. Qualitative (n=22) and mixed methods (n=9) designs were widely represented. We identified the following 7 themes that influenced user engagement: personalization (n=29), reinforcement (n=23), communication (n=20), navigation (n=17), credibility (n=16), message presentation (n=16), and interface aesthetics (n=7). A checklist was developed that contained these 7 design features and 29 corresponding specific implementations derived from the studies.

**Conclusions:**

This systematic review and thematic synthesis identified useful design features that make an mHealth intervention more user friendly. We generated a checklist with evidence-based items to enable developers to use our findings easily. Future evaluations should use more robust quantitative approaches to elucidate the relationships between design features and user engagement.

## Introduction

Mobile health (mHealth) is a means of providing medical and public health support to health care consumers via mobile devices, such as mobile phones, portable computers, and personal digital assistants [1]. mHealth interventions involve the adoption of mobile technologies to provide educational information, help users manage their own conditions and behaviors, and deliver health care to improve the health of users. Compared to traditional delivery models, mobile interventions can be more cost-effective [2,3]; help users overcome demographic, socioeconomic, and geographic barriers to access [4-6]; allow the privacy of users to be protected [7]; and allow a high level of customization, self-management, and communication [8-11]. With the popularity of smartphones, mHealth technology has grown rapidly in the past 10 years and has been used in many health fields. Mobile interventions have been shown to improve healthy behaviors (eg, weight loss [12], balanced diet consumption [13], and smoking cessation [14]) and disease management [15] (eg, tuberculosis [16] and AIDS [17]).

mHealth programs require autonomous use [5], which depends on user involvement and self-management, and engagement is related to behavior changes and health improvements [18]. User engagement refers to high uptake, high-quality user experience, and good adherence over long periods of time [19]. However, a pressing concern regarding mHealth interventions is the high reported rate of disengagement [4]. For example, in a web-based weight loss study, respondents did not use the app as intended, and only 64% of the intervention group actually used the intervention at least once [20]. A mobile phone text message smoking cessation program also suffered from a high attrition rate: nearly half of the subscribers did not complete the entire program in the real-world implementation phase, and the majority of opt-outs occurred in the first 2 weeks [21]. With regard to web-based physical activity interventions, reported dropout attrition rates vary between 0% and 62% [22,23]. This is a common issue related to mHealth interventions; poor user engagement makes intervention effectiveness difficult because the users are not exposed to enough of the intervention content [24].

The development steps of an mHealth intervention tool can influence user engagement. The first step is intervention content development. User engagement will be low if the content does not adhere to what has been shown to be effective [25,26], and there are already well-established guidelines for health intervention content development [25,27,28]. The second step is design of the mode by which the intervention content is delivered (eg, information architecture, screen appearance, and interactive features). Poor design features, such as complicated navigation and difficult-to-read screen presentations, are poorly tolerated by users in real-world settings [29]. Good mHealth design is readily distinguishable from its competitors, leading users to feel more favorably disposed toward the product and have a positive user experience [4,19,30,31]. Several studies analyzed which design features should be included in mHealth interventions [32-35]; however, they did not clearly describe how to specifically deliver the interventions, and the contributions of most of the design features mentioned have not been tested in empirical studies. In addition, Morrison et al [28] developed a hypothetical framework to define the design features through a review, but the framework only focused on 4 interactive design features (social context and support, contact with the intervention, tailoring, and self-management) and provided simple definitions of the other 8 features that were difficult to implement in subsequent studies. Webb et al [36] developed a coding scheme for design features in a meta-analysis; however, this scheme was proposed before a review of the literature was performed rather than derived from the literature reviewed, so it inevitably missed features that are important but not reported.

While these studies have provided some important guidance for the design features of mHealth interventions, none has included comprehensive design features based on the literature or experiences. Additionally, except for Crutzen et al [34], other researchers did not focus on the relationships between the design features mentioned and user engagement. Furthermore, the data on which these studies were based were obtained more than 10 years ago, and in the past 10 years, the popularity of mHealth interventions has rapidly expanded due to the use of smartphones; data from more recent studies need to be considered. The objectives of our study were to systematically review studies published in the past 10 years regarding design features that improve user engagement with mHealth interventions and generate a checklist that can easily be used during the design of future mHealth interventions.

## Methods

### Protocol

This systematic review followed the PRISMA (Preferred Reporting Items for Systematic Reviews and Meta-Analyses) checklist [37]. The protocol was registered in the International Prospective Register of Systematic Reviews (CRD42020140282).

### Inclusion and Exclusion Criteria

Articles were selected if they met the following criteria: (1) The study was empirical. The study population was composed of users or potential users of mHealth. If a study focused on special populations, such as children and older adults, it was excluded. (2) The study focused on the mode of delivery of health intervention content via mobile devices, for example, aesthetics, message phrasing, and interactive features. Articles that mentioned design features but did not explain them, making it unclear which features were being referenced, were excluded. Studies on the selection of intervention content or theory, and those providing a general description of the process of designing an mHealth intervention were excluded. (3) The study reported quantitative and qualitative analyses of the effectiveness of the design features with regard to increasing user engagement or user acceptance of the design features. (4) The article was published in a peer-reviewed journal from January 1, 2009 to June 13, 2019. (5) The article was published in English or Chinese.

### Search and Screening Strategy

Five databases, namely, PubMed, Web of Science, Cochrane Library, Ovid EMBASE, and Ovid PsycINFO, were searched in June 2019. The search strategy was *“mobile health”* or *mHealth* or *m-health* or *eHealth* or *e-health* combined with design or feature* or principle* or “mode* of delivery” or *model* and combined with engage* or adhere* or maintain* or retention or sustain* or usage* or satisf* or *prefer* or preference* or accept* or *reliable*.

**Figure 1:**
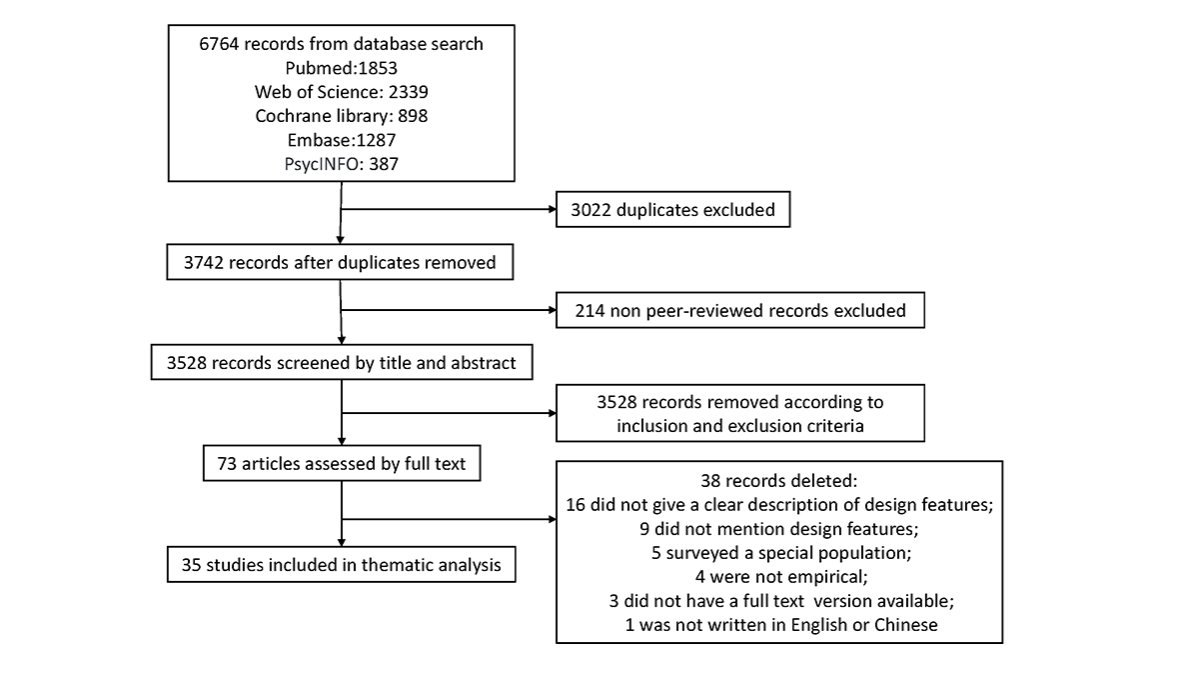
Screening flowchart.

The search results were uploaded to EndNote (Version X9; Clarivate Analytics) for screening. Figure 1 shows the process of identifying the eligible articles. Duplicates were identified with the sorting function in EndNote. Peer-reviewed journal articles were checked by searching Ulrich's Periodicals Directory; some journals could not be found there, so we visited the journal website to find evidence of the peer-review process. All remaining articles were assigned to 1 of 3 reviewers and were screened by titles, abstracts, and then full texts according to inclusion and exclusion criteria. YW screened all journal articles independently again. Any disagreement was discussed among the reviewers.

### Data Extraction and Synthesis

All selected articles were imported into NVivo (version 11; QSR International). The following study characteristics were extracted: (1) article characteristics, including country and year of publication; (2) health topic; (3) participant characteristics, including sampling methods, sample size, sex, and age; (4) data collection method; and (5) mobile technology. Study characteristics were analyzed using descriptive statistics.

The thematic synthesis analysis method developed by Thomas and Harden [38] has 3 steps: (1) line-by-line coding of the articles to record the components, (2) the development of descriptive themes, and (3) the creation of analytical themes. We performed the first 2 steps together. YW coded each line of the records according to its meaning, translated the concepts among the records, and developed the descriptive themes. HD validated the results by comparing each assigned code to the full texts of the articles. Then, analytical themes were developed by answering the review question using the existing descriptive themes. Each reviewer did this independently, and the results were discussed among all authors. The coding process was iterative.

### Assessing Study Quality

The Mixed Methods Appraisal Tool (MMAT) [39] was used to assess the methodological quality of the included studies, including the data collection methods, participant sampling, interpretation of results, consideration of confounders, and risk of bias. In the MMAT, there are 5 criteria with responses “yes,” “no,” and “cannot tell” for each research design. The retained studies were assessed, and the results recorded by 2 authors (YW and HD), independently.

## Results

### Study Characteristics

All 35 articles included in the analysis were published between 2011 and 2019. They were primarily from the United States (n=14), the United Kingdom (n=9), and Australia (n=5). In terms of the health topics discussed in these studies, 17 articles focused on unhealthy lifestyles (eg, smoking, excessive alcohol consumption, sleep disturbances, and poor sexual health), 10 studies focused on chronic diseases (eg, diabetes, breast cancer, chronic arthritis, and asthma), 4 studies focused on mental health problems, and 4 studies focused on other health issues. Qualitative studies (n=22) and mixed methods (n=9) were the most common data collection methods used, accounting for 89% (31/35) of all studies. The sample sizes ranged from 8 to 1865 in the 35 studies, and the age of the participants ranged from 14 to 74 years old. The mobile technology used in the majority of studies was a mobile phone app (n=24), followed by a website platform (n=6), and text messages (n=5). More detailed information is presented in Multimedia Appendix 1.

### Study Quality

A total of 3 articles meet the criteria of all 5 items, with the remaining articles meeting 4 criteria (n=18), 3 criteria (n=13), and 2 criteria (n=1). The most common reasons low scores in each research design were a lack of coherence between the data collection and analysis and the explanations in qualitative studies; the quality of different components was low in the mixed methods studies; and there were poor sampling strategies and a high risk of bias in the quantitative studies (Multimedia Appendix 2).

### Themes of Design Features

**Figure 2:**
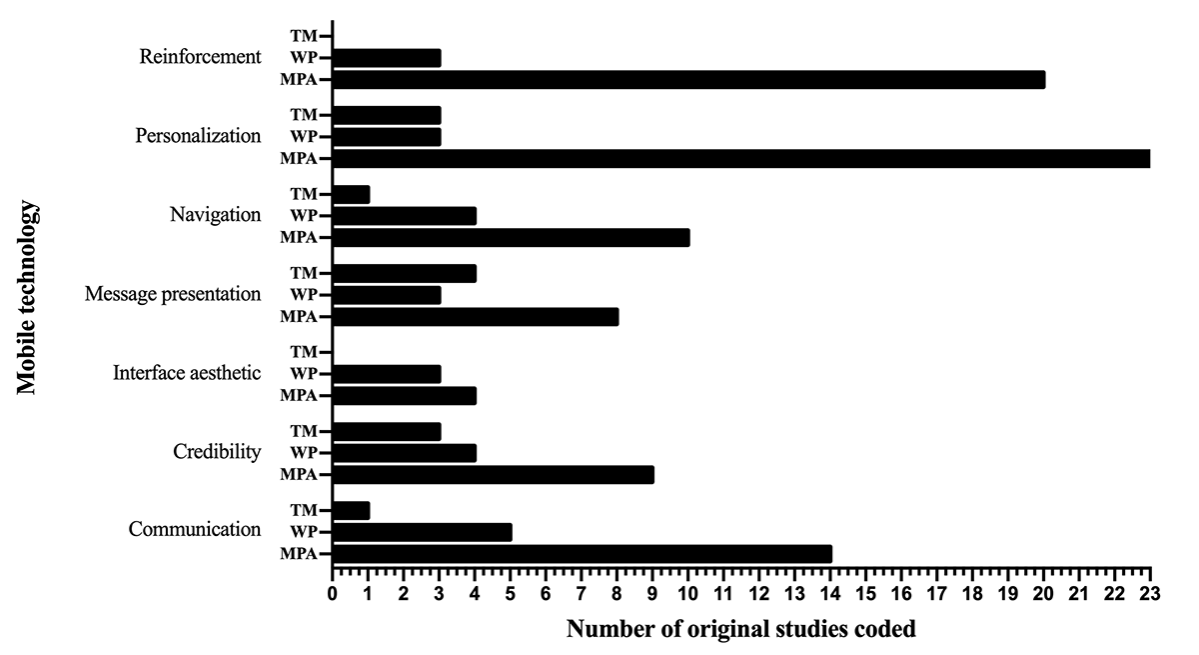
Comparison of analytical themes in different mobile technologies. TM: text message; WP: website platform; MPA: mobile phone app.

**Figure 3:**
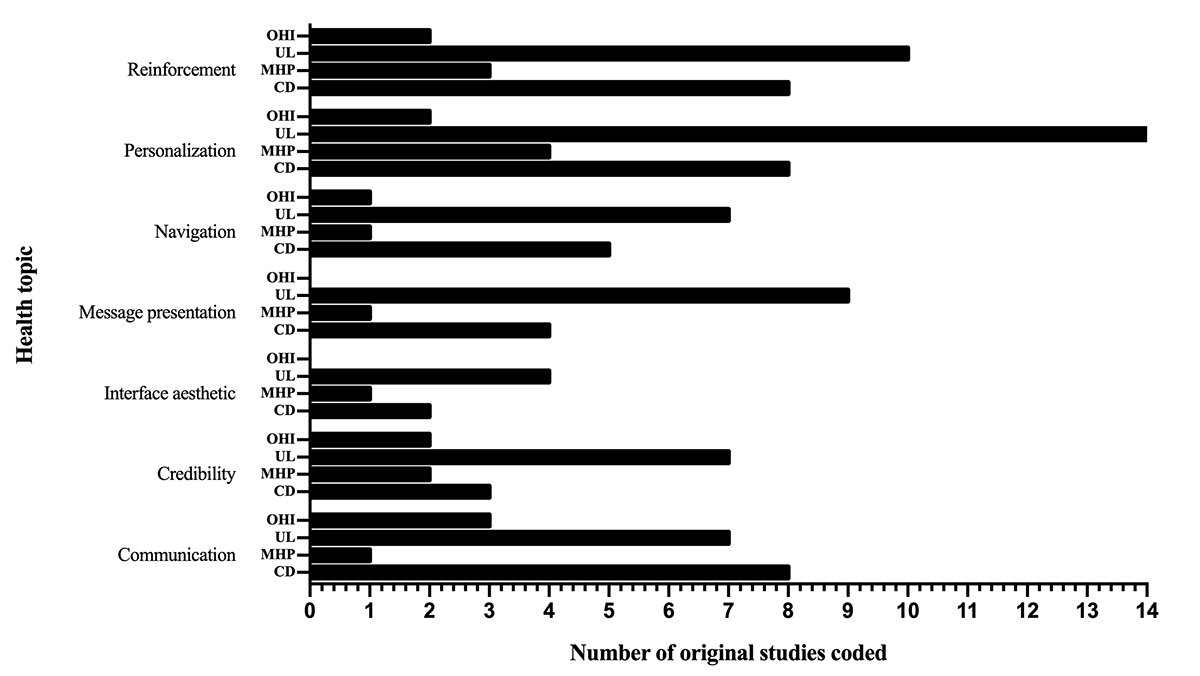
Comparison of analytical themes in different health topics. OHI: other health issues; UL: unhealthy lifestyle; MHP: mental health problem; CD: chronic disease.

In total, 7 analytical themes were generated to describe the design features that can improve user engagement with mHealth interventions, and each of these can be explained by several descriptive themes. With regard to the 3 types of mobile technology used in the studies, *mobile phone apps* and *website platforms* share common design features, while *text messages* lack 2 analytical themes: interface aesthetic and reinforcement (Figure 2). For health topics, *unhealthy lifestyles*, *chronic diseases*, and *mental health problems* share common design features, and the topic of *other health issues* lacks interface aesthetic and message presentation (Figure 3).

### Interface Aesthetic

#### Overview

The interface refers to the appearance of the screen, which was reported to directly impact the user's impression of the intervention and impact user engagement in 7 of the 35 studies (20%). Two descriptive themes that could improve interface aesthetics were identified: (1) attention-grabbing and (2) simple and consistent style.

#### Attention Grabbing

An aesthetically appealing screen easily attracts user attention [40]. Users preferred that the screen show graphics rather than too much text because the latter can be overwhelming [41,42]. They also preferred a pleasing color scheme. Bright colors (eg, light green, white) were considered attention grabbing, while dark and neon colors discouraged further use [43,44].

#### Simple and Consistent Style

A simple and clean screen was praised most frequently in the studies, while users disliked complex and overcrowded pages, which rapidly made them lose interest [40,41,44,45]. Many users appreciated the use of a consistent style, with a coherent presentation in terms of colors, pictures, and themes throughout an mHealth intervention [41,46].

### Navigation

#### Overview

Navigation describes how users move to different areas of content within mHealth intervention apps. There were 2 descriptive themes pertaining to navigation: (1) ease of use and (2) automation; these themes were proposed by users in 17 of the 35 studies (49%).

#### Easy to Use

The users highlighted the importance of minimum input and efficient access to information, such as a simple log-in process, fewer required tasks, or fewer buttons on the screen [43-53]. An interactive process that confused the users or that took them many iterations to understand prevented them from continuing to engage with the app. Explanation of how the mHealth intervention worked (ie, clarifying what to do next) promoted continued use [41,50,53,54].

#### Automation

Users did not want to spend much time scrolling to find the information they wanted. The search bar and menu bar, which provide options to the user, were thought to facilitate usage [44,46,55-58].

### Personalization

#### Overview

Personalization is a design feature that makes mobile technology act in a particular way depending on user preferences. Personalization was achieved by the following 3 elements according to 29 of the 35 studies (83%): (1) assessment, (2) feedback, and (3) manipulation.

#### Assessment

Users expected to be assessed with metrics pertaining to the health problem that was the focus of the intervention in as much detail as possible to create an accurate profile [49,59-61]. Some parameters, such as sociodemographic characteristics, basic health status, and individual preferences and habits, could be measured with a quick survey within an app [54,55,57,62,63]. Users also liked continuous monitoring features that allowed them to record their progress toward their goal on a daily basis (eg, health and behavior changes and adherence to an intervention) [40,42,43,45,58,64-67] or diary entries or notes that helped them track their progress [40,45,60]. Moreover, users also indicated a preference for sensor-based automated tracking as opposed to self-reported data, which they often forgot to input and found were not as convenient [47,52,59,63,68].

#### Feedback

Studies indicated the importance of building an assessment on the basis of feedback on the acquired data; users quickly lost interest when they did not receive feedback that was customized [41,69]. The preference was for the mobile device to provide personalized information, including tailored intervention content matched to their basic characteristics [40,43,45,46,51,54,55,57-65,67,68,70,71] and feedback on continuous monitoring data, for example, their health and behavior progress over time, predicted possible causes and consequences of a health problem and advice on the behavior under investigation [41-43,47,49,52,53,58,59,63,68]. There was a strong interest among users for visualization of continuous monitoring data, for example, presenting data as graphs and tables [47,49,53,58,63,67,69].

#### Manipulation

Users not only wanted to obtain automatically tailored information but also wanted to be able to customize the mHealth intervention themselves. Users highlighted the importance of being able to choose when and how they receive reminders [43,45,53,57,63-65,72], set goals for the future use of the tool [43,47,49,53,54,58,63,65], and select preferred styles, such as the color and font [46,54,59].

### Reinforcement

#### Overview

Reinforcement is the provision of a stimulus to strengthen the likelihood of a user continuing to exhibit a certain behavior in the future. There were 2 descriptive themes extracted from 23 of the 35 studies (66%), namely, (1) rewards and (2) reminders, that helped enhance reinforcement.

#### Rewards

The reward feature could increase user motivation to engage with the intervention, and users expressed desire for confirmation when they completed a task. The reward features extracted from the studies included material incentives (eg, cash or gifts), intangible rewards (eg, virtual badges, rankings, certificates, and points), and messages of congratulations when a task was completed [40,41,43,49,51-54,59-61,63].

#### Reminders

Users wanted reminders to schedule a task, such as taking medicine, making and keeping clinic appointments, and continuing a health plan [49,55,58,60,63,66,68]; to return to their mobile device [42,46,64,70]; and to motivate them with information and advice [40,45,52,61,67,72]. The preferred forms of reminders included email messages, text messages, words of the day, and pop-ups [45,49,52,61,70].

### Communication

#### Overview

Communication is a function that prompts users to consult and communicate with other people regarding their health problems via a mobile device. (1) Peer-to-peer communication and (2) access to professionals were 2 aspects of communication reported in 20 of the 35 studies (57%).

#### Peer-to-Peer Communication

The users expressed interest in communicating with other people with similar experiences through online forums, communities, by instant messages within an mHealth tool, or by connecting via other types of social media; they wanted to be able to post information, share their stories, ask and answer questions, and find mutual accountability partners [40,45,46,54-56,58-60,63,66,67,69,72].

#### Access to Professionals

Users wanted to be able to directly contact a health care provider via email, text message, or live chat to ask them questions or obtain advice based on their health data [40,45,48,53-56,58-61,64,66,68,72].

### Message Presentation

#### Overview

The presentation of information is an important factor that impacts user engagement, and information that is presented well is readily accepted by users. (1) Language, (2) tone of voice, and (3) presentation design are points of consideration when seeking to improve message presentation, according to the results of 16 of the 35 studies (46%).

#### Language

The message needs to be clearly presented in the language used by the audience. The users recommended using simple nontechnical language that is straightforward and concise; they were tired of patronizing and technical language [48,55,62,69,73,74]. When providing an actionable message, users preferred a specific description that made it clear what they were supposed to do [70,71,73].

#### Tone of Voice

Users recommended using language that framed the information positively rather than negatively, as the latter made them feel discouraged and made them want to turn off the device [43,51,54,62,71,73,74]. To increase user acceptance, it was essential to make the user feel supported and relaxed by using a nonauthoritarian, friendly, and nonjudgmental tone of voice [41,45,69,71].

#### Presentation Design

The users were quickly bored with text-heavy presentations of information; they wanted multimedia messages, for example, text combined with relevant pictures or video [44,46,53,74]. Knowledge quizzes and games were also recommended as ways to deliver information that prompted the user to engage and learn the information [44,54]. The use of various font styles, sizes, and colors to highlight key information was suggested, as it allowed the user to skim quickly when they lacked the patience to read the entire message [44,46]. Editing the text to make it as concise as possible was also suggested by users, who were not inclined to read lengthy messages [53,62,71].

### Credibility

#### Overview

Credibility is an important feature that guarantees the level of user comfort, enabling them to engage with the mobile technology without experiencing concerns. (1) Trustworthiness and (2) confidentiality were 2 descriptive themes related to credibility derived from 16 of the 35 studies (46%).

#### Trustworthiness

Users trusted mHealth interventions from authoritative and familiar organizations or developers that were free from advertisements [41,43,48,54,61,63,68,74]. Users emphasized the fact that the information provided needed to be evidence-based and from credible sources to gain their trust [50,57,61,62,65,69].

#### Confidentiality

Users highlighted the importance of having a privacy policy, for example, a policy that allowed the users to decide whether others could access their data [50,56], ensured that the users remained anonymous when sharing their data with the health care providers or for research [43,64], and allowed users to set passwords for protection [45,61].

### Checklist of Design Features to Enhance User Engagement

Based on these themes, we produced a checklist that considers 7 aspects of design and the corresponding implementations based on an exhaustive analysis of the 35 studies. In total, there were 29 items reported that enhance user engagement, and we provide here the descriptions and examples as a reference for future studies (Table 1).

**Table 1:** Checklist of design features that enhance user engagement.

Items			Criteria
**Interface aesthetic**			
	1	The screen shows a graphic presentation rather than too much information	
	2	Pleasing color scheme with bright colors (eg, light green, white)	
	3	Simple screen presentation that is not overcrowded	
	4	Coherent scheme of colors, pictures, and themes throughout the intervention	
**Navigation**			
	5	Minimum user input needed; efficient access to the information provided, such as in a simple menu; and few buttons on the screen	
	6	Guidance provided that explains how the mHealth intervention works	
	7	Search bar or menu bar provided to accelerate the process of finding certain information	
**Personalization**			
	8	Assessment of the preferences, sociodemographic characteristics and health status of the user	
	9	Continuous monitoring of health and behavior changes or adherence to an intervention	
	10	Provision of a diary or note-taking function	
	11	Provision of personalized information matched to the user's characteristics	
	12	Provision of feedback on the continuously monitored data	
	13	Visual presentation of feedback, such as in graphs and tables	
	14	Provision of autonomy to customize the intervention, for example, allowing the users to choose when and how they receive reminders, to set a goal about their future use of the intervention tool, and to select their preferred styles, such as their preferred colors and fonts	
**Reinforcement**			
	15	Provision of material incentives (eg, cash or gifts), intangible rewards (eg, virtual badges, rankings, certificates, and points), or messages of congratulations when a task is completed	
	16	Sending of reminders to facilitate the scheduling of tasks and to ensure continuous use	
**Communication**			
	17	Provision of access to other people with similar experiences through an online forum, community, or instant messages within the mobile tool or by connection with other forms of social media	
	18	Provision of access to a health care provider through email, text message, or live chat	
**Message presentation**			
	19	Use of simple nontechnical language that can be readily understood	
	20	Use of specific descriptions when providing actionable message	
	21	Use of a positive, nonauthoritarian, and nonjudgmental tone of voice	
	22	Provision of multimedia messages, for example, text combined with relevant pictures or videos	
	23	Presentation of information in the form of knowledge quizzes and games, if possible	
	24	Use of various font styles, sizes, and colors to highlight information	
	25	Editing of the text to make it as concise as possible	
**Credibility**			
	26	Absence of advertisements	
	27	Provision of evidence-based information from credible sources	
	28	Provision of a privacy policy that gives users the right to decide whether others can access their data and ensures the users remain anonymous when sharing their data with the health care providers or for research	
	29	Enabling users to set a password or code to protection their data	

## Discussion

### Overall Findings

We used thematic synthesis to identify design features that increased user engagement with mHealth interventions based on user feedback. For each design feature, specific implementations in mobile tools were also analyzed. This study presented 7 analytical themes and 16 descriptive themes pertaining to design features that can improve user engagement with mHealth interventions. From most to least commonly mentioned in the studies, the analytical themes were personalization (29/35, 83%), reinforcement (23/35, 66%), communication (20/35, 57%), navigation (17/35, 49%), credibility (16/35, 46%), message presentation (16/35, 46%), and interface aesthetic (7/35, 20%); each analytical theme involves several descriptive subthemes that explain how to implement them when designing mHealth interventions. Overall, the 7 analytical themes were applicable to different mobile technologies and health topics, indicating that the design features identified by this study are universal across mobile apps, website platforms, text messages, and different health themes. 

To promote better application of the results of this study to future mHealth intervention development, we developed a checklist of the design features that enhance user engagement; this tool has 29 evidence-based items that are clearly described to make them easy to use by developers of mHealth interventions.

Personalization, reinforcement, and communication were the design features that were mentioned the most often. Compared to other analytical themes, these 3 design features focused on the interactivity of mHealth interventions, including user-to-technology interactions and user-to-user interactions. User-to-technology communication refers to having the user input information about themselves to which the tool provides a tailored response [75]. Two analytical themes, personalization and reinforcement, pertain to the interaction between users and technology. User-to-user interactions are represented by the theme of communication in this article, including peer-to-peer contact and consultation with professionals. Interactive features give users a sense of ownership [76] and promote their participation in the mHealth intervention. Compared with traditional smoking cessation methods, a major value of mobile health interventions is that they can provide better and faster interactions [77] to meet the needs of users.

Personalization, reinforcement, and communication are design features of mobile health interventions and behavior change techniques [25] that can improve the effectiveness of the interventions. Morrison et al [28] also showed that personalization and communication are related to effective intervention outcomes. Therefore, these interactive design features can improve user participation and promote the effectiveness of interventions, and special attention should be given to them during the design of mobile health interventions.

In terms of presentation and navigation, users preferred a user friendly design, specifically, one that was easy to use and understand and was aesthetically pleasing, which was mainly addressed by the design of the interface aesthetic, navigation, and message presentation. A user friendly design is easy to use and understand, with features such as simple and convenient navigation, easy to understand language, and a supportive tone. Users often experience difficulties when using new technology [78], and an easy-to-use design can reduce that burden [79,80]. A study [41] showed the importance of a simple design when engaging users who were resistant to change. Compared to users who intend to change, those who were resistant to change were harder to engage in an intervention; however, this issue was alleviated with the help of a simple design [81].

Aesthetics also increases the friendliness of the design. A lack of aesthetics and text-heavy presentations made users feel bored, and users liked information presented with pictures and short texts. Additionally, a beautiful interface more easily attracts the user's attention. Studies have shown that a friendly mobile health intervention design can win the trust of users [82] and that credibility is a major concern for potential users [83]. Credibility needs to be established for the user to trust and use a mobile tool [84]. Tools that are developed in the future should provide evidence-based information, privacy policies, and password protection; they should also remove advertisements.

The 7 analytical themes from this study all appeared in different health topics, indicating that the design features proposed in this study were universal across the different health topics. Mobile apps and the website platforms shared 7 analytical themes, but text messages lacked reinforcement and interface aesthetics. The reason for this may be that the small number of articles involving text messages and the features designed to improve user engagement were not comprehensive and that text messages can be regarded as simple mobile apps, which cannot realize all of the functions of mobile apps; for example, the aesthetic interfaces design feature does not apply to text messages.

Mobile phone apps and website platforms are currently the most advanced mHealth technologies [85]. From this perspective, the design features of this study are also universal across different mobile technologies. Other simpler technologies can select a part of the design features in the checklist as a reference according to their own functions. Different mHealth technologies have different capabilities for presentation, navigation, and interaction, which may lead to different potential for improving user engagement. In the future, the relationship between different mHealth technologies and user engagement needs to be studied.

One study [86] about the design features of 100 smoking cessation apps on iTunes in 2016 showed that the existing smoking cessation apps performed well in terms of language but performed poorly in terms of presentation, navigation, and interaction. Our design feature checklist can help solve this problem. Researchers can use the checklist to guide the design process of mHealth interventions. The checklist can also be used to evaluate mHealth interventions that have been developed.

There is no unified terminology for design features. The same design feature has multiple different names in different studies. For example, communication can also be called *social support* and *social network*. This study summarized and translated the existing descriptions and described each design feature in a unified term to promote a standardized description of the design features.

### Limitations

This study has the following limitations. First, we only selected studies that explained how a design feature is implemented in technology because some articles lacked detailed descriptions, making them impossible to analyze. However, this may mean that the design features we extracted do not comprehensively represent all research that has been performed. Second, most included articles were qualitative and mixed methods studies, which have considerable advantages given the exploratory nature of this research; however, compared with quantitative research, these methods provide less convincing evidence of the relationship between design features and user engagement. There may be sufficient studies available to explore design features that can improve engagement, and a more robust quantitative study design is needed to verify the association. Third, the mobile technology used in the studies in this review were mobile phone apps, website platforms, and text messages, which do not represent all technologies used in mHealth interventions. Other mobile tools, such as remote measurement technology and wearable devices, do not have as many functions as the aforementioned technologies included in this study. Subsets of the design features proposed in our research can also be applied to other forms of mobile technology, and relevant studies, especially regarding remote and wearable devices, are needed, considering their differences compared with the mobile phones, tablets and laptops used in this research. Fourth, the MMAT is currently the most applicable appraisal tool for a systematic mixed studies review, and it provides a detailed assessment of the quality of the included studies. The quality of the included studies varied, and 14 articles met 2 or 3 criteria of all 5 items, which means bias is present. However, the 2018 version MMAT does not provide quantification of a study’s quality, and due to the heterogeneity of the included studies and the qualitative design of this systematic review, it is difficult to assess the weight of the different included studies or to obtain a more accurate understanding of the overall risk of bias.

### Conclusions

This study summarized research results obtained in the past ten years to identify design features in mHealth interventions that improve user participation. We made a checklist that divided the design features of mHealth interventions into 7 different aspects with associated, clearly described implementations, which can not only be used as a reference during the mHealth development process but also as an evaluation tool for the design features of newly developed mHealth interventions. This checklist can be applied to mobile apps, website platforms, and text messages and can be applied to health topics such as unhealthy lifestyles, chronic diseases, and mental health problems. The study of the relationships between these design features and user engagement is in the exploratory stage but has great potential. We synthesized the results of currently available studies to promote better application of their results and to lay a foundation for subsequent confirmatory research.

## Appendix

Multimedia Appendix 1 Characteristics of the original studies.

Multimedia Appendix 2 Quality assessment methodology.

## Abbreviations

mHealth: mobile health
MMAT: Mixed Methods Appraisal Tool
