# Supplementary material for: Design Features for Improving Mobile Health Intervention User Engagement: Systematic Review and Thematic Analysis
Source: J Med Internet Res. 2020 Dec 9;22(12):e21687. doi: 10.2196/21687 (PMC7758171; doi:10.2196/21687)
Supplement: Multimedia Appendix 1 [file jmir_v22i12e21687_app1.doc]

| Articles | Year of publication | Country | Health topic | Participants | | | | Data collection methods | Mobile technologies |
| --- | --- | --- | --- | --- | --- | --- | --- | --- | --- |
|  |  |  |  | Sampling method | Sample size | Sex | Age (years) |  |  |
| Utrankar A, et al | 2018 | The United States | Sickle cell disease | Convenience sampling | Focus group (N=39) and survey/interview (N=47) | Focus group (77% female) and survey/interview (53% female) | Focus group: median (range)=45 (25–66); Survey/interview: median (range)=28 (14–61) | Mixed methods | Mobile phone application |
| Coyne I, et al | 2016 | Ireland | Chronic disease | Not reported | Postal survey (N = 207) and interviews (N = 21) | Not reported | 14–25 | Mixed methods | Website platform |
| Grunberg PH, et al | 2018 | Canada | Infertility | Convenience sampling | 519 | 55% female | Mean± standard deviation=36 ± 6 | Cross-sectional design | Website platform |
| Gatwood J, et al | 2019 | The United States | Diabetes | Purposive sampling | 16 | 81% female | Range=45-70 | Focus group (2) | Text message |
| Thornton LK and Kay-Lambkin FJ | 2018 | Australia | Mental health problems | Not reported | 284 | 71% female | Mean± standard deviation=31±15 | Cross-sectional design | Mobile phone application |
| Jennifer B McClure, et al | 2013 | The United States | Smoking | Not reported | 1865 | 63% female | Mean± standard deviation=44 ± 15 | Randomized Factorial Trial | Mobile phone application |
| Solem IKL, et al | 2019 | Norway | Chronic pain | Not reported | 20 | 85% female | Median (range)=48 (18-74) | Semi-structured interview | Information and communication technology |
| Peng W, et al | 2016 | The United States | Type 2 diabetes | Not reported | 18 | 72% female | Mean± standard deviation=54 ±13 | Focus group (4) | Mobile phone application |
| Rabin C, Bock B. | 2011 | The United States | Lack of exercise | Not reported | 15 | Not reported | Mean± standard deviation=39± 13 | Mixed methods | Mobile phone application |
| Willoughby JF, Muldrow A. | 2017 | The United States | Poor sexual health | Not reported | 31 | 68% female | 20 ±2 | Mixed methods | Text message |
| Perski O, et al. | 2018 | United Kingdom | Excessive alcohol consumption | Not reported | Focus group (N=9) and online survey (N=132) | Focus group (78% female) and online survey (49% female) | ≥18 | Mixed methods | Mobile phone application |
| Saberi P, et al. | 2016 | The United States | AIDS | Not reported | 17 | 12% female | Mean± standard deviation=25± 3 | Focus group (4) | Mobile phone application |
| Evans C, et al. | 2016 | United Kingdom | Poor sexual health | Purposive sampling | 48 | 60% female | Range=18-45 | Focus group (6) | Text message |
| Crane D, et al. | 2017 | United Kingdom | Excessive alcohol consumption | Purposeful sampling | Think aloud (N=12) interview (N=12) | Think aloud (50% female} and interview (50% female) | Think aloud (mean=42) and interview (mean=40) | Qualitative study (focus group and individual interview) | Mobile phone application |
| Gkatzidou V, et al. | 2015 | United Kingdom | Poor sexual health | Convenience sampling | 49 | 55% female | Median=19 | Focus group (9) | Mobile phone application |
| Peng W, et al | 2016 | The United States | Not limited to one topic | Purposive sampling | Focus group (N=39) and individual interview (N=5) | 65% female | ≥ 18 | Qualitative study (focus group and individual interview) | Mobile phone application |
| Perski O, et al. | 2017 | United Kingdom | Smoking and excessive alcohol consumption | Convenience sampling and snowballing sampling | 20 | 60% female | Mean± standard deviation=30± 9 | Qualitative study (think aloud and interview) | Mobile phone application |
| Zhao Y, et al. | 2018 | China | Poor sexual health | Not reported | 36 | 0% female | ≥18 | In-depth interview | Text message |
| Fylan F, et al. | 2018 | United Kingdom | Not limited to one topic | Not reported | 55 | Not reported | Not reported | Focus group (8) | Mobile phone application |
| Lyzwinski LN, et al. | 2018 | New Zealand | Mental health problems | Purposive sampling | 8 | Not reported | ≥18 | Qualitative study (focus group and email feedback) | Mobile phone application |
| Phillips SM, et al. | 2019 | The United States | Lack of exercise | Not reported | Online questionnaire (N= 96) and interview (N=28) | 100% female | Online questionnaire: mean± standard deviation=56±10; interview: mean± standard deviation=53±10 | Mixed methods | Mobile phone application |
| Herbec A, et al. | 2018 | United Kingdom | Smoking | Not reported | 16 | 50% female | Range= 20-51 | Qualitative study (interview and think aloud) | Mobile phone application |
| Goldenberg T, et al. | 2014 | The United States | Poor sexual health | Not reported | 38 | 0% female | Mean (range)=32 (19-63) | Focus group (5) | Mobile phone application |
| Hilliard ME, et al. | 2014 | The United States | Cystic fibrosis | Not reported | 15 | 47% female | Mean± standard deviation=30±6 | Mixed methods | Mobile phone application |
| Milward J, et al. | 2017 | United Kingdom | Excessive alcohol consumption | Not reported | 20 | 90% female | Mean± standard deviation=23±4 | Focus group (3) | Mobile phone application |
| Lazard AJ, et al. | 2019 | The United States | Smoking | Not reported | 39 | Not reported | Mean± standard deviation=19±3 | Focus group (4) | Website platform |
| Su MC, et al. | 2015 | Taiwan | Metabolic syndrome | Snowballing sampling | 20 | 100% female | Mean=18 (16-20) | Individual interview | Website platform |
| Hartzler AL, | 2016 | The United States | Smoking | Purpose sampling | 40 | 50% female | Mean± standard deviation=38±12 | Mixed methods | Mobile health technologies |
| Geuens J, et al | 2019 | Belgium | Chronic arthritis | Purposive sampling | 31 | 45% female | Mean± standard deviation=51± 12 | Mixed methods | Mobile phone application |
| Peters D, et al. | 2018 | Australia | Mental health problems | Not reported | 60 | 8% female | Mean (range)=47(26-66) | Qualitative study (participatory workshops) | Mobile phone application |
| Carolan S and de Visser RO | 2018 | United Kingdom | Mental health problems | Not reported | 18 | 78% female | Mean± standard deviation=45±10.8 | Individual interviews | Web-based platform on computer or smartphone |
| Aji M, et al. | 2019 | Australia | Sleep disturbances | Not reported | 9 | 33% female | Range=21-70 | Focus group (2) | Mobile phone application |
| Peters D, et al | 2017 | Australia | Asthma | Convenience sampling | 20 | 60% female | Mean (range)=18 (15-24) | Qualitative study (participatory workshop) | Mobile phone application |
| McClure JB, et al. | 2017 | The United States | Smoking | Not reported | 116 | 72% female | Mean± standard deviation=38 ±12 | Quantitative description | Mobile phone application |
| Wright CJC, et al. | 2017 | Australia | Excessive alcohol consumption | Not reported | 42 | 50% female | Range= 18-25 | Qualitative study (workshop) | Text message |
