# Supplementary material for: Design Features for Improving Mobile Health Intervention User Engagement: Systematic Review and Thematic Analysis
Source: J Med Internet Res. 2020 Dec 9;22(12):e21687. doi: 10.2196/21687 (PMC7758171; doi:10.2196/21687)
Supplement: Multimedia Appendix 2 [file jmir_v22i12e21687_app2.doc]

| Articles | Qualitative study | | | | | Mixed methods study | | | | | Quantitative non-randomized controlled trial | | | | | Quantitative randomized controlled trial | | | | | Quantitative descriptive study | | | | |
| --- | --- | --- | --- | --- | --- | --- | --- | --- | --- | --- | --- | --- | --- | --- | --- | --- | --- | --- | --- | --- | --- | --- | --- | --- | --- |
|  | Item1 | Item 2 | Item 3 | Item 4 | Item 5 | Item 1 | Item 2 | Item 3 | Item 4 | Item 5 | Item 1 | Item 2 | Item 3 | Item 4 | Item 5 | Item 1 | Item 2 | Item 3 | Item 4 | Item 5 | Item 1 | Item 2 | Item 3 | Item 4 | Item 5 |
| Utrankar, et al |  |  |  |  |  | 2 | 1 | 1 | 1 | 2 |  |  |  |  |  |  |  |  |  |  |  |  |  |  |  |
| Coyne, et al |  |  |  |  |  | 1 | 1 | 2 | 1 | 2 |  |  |  |  |  |  |  |  |  |  |  |  |  |  |  |
| Grunberg, et al |  |  |  |  |  |  |  |  |  |  | 2 | 1 | 2 | 1 | 1 |  |  |  |  |  |  |  |  |  |  |
| Gatwood, et al | 1 | 2 | 2 | 1 | 1 |  |  |  |  |  |  |  |  |  |  |  |  |  |  |  |  |  |  |  |  |
| Thornton and Kay-Lambkin |  |  |  |  |  |  |  |  |  |  | 2 | 1 | 2 | 1 | 1 |  |  |  |  |  |  |  |  |  |  |
| Jennifer, et al |  |  |  |  |  |  |  |  |  |  |  |  |  |  |  | 2 | 1 | 1 | 2 | 1 |  |  |  |  |  |
| Solem, et al | 1 | 1 | 2 | 1 | 1 |  |  |  |  |  |  |  |  |  |  |  |  |  |  |  |  |  |  |  |  |
| Peng, et al | 1 | 1 | 1 | 1 | 2 |  |  |  |  |  |  |  |  |  |  |  |  |  |  |  |  |  |  |  |  |
| Rabin. |  |  |  |  |  | 1 | 1 | 2 | 1 | 2 |  |  |  |  |  |  |  |  |  |  |  |  |  |  |  |
| Willoughby. |  |  |  |  |  | 1 | 2 | 2 | 1 | 2 |  |  |  |  |  |  |  |  |  |  |  |  |  |  |  |
| Perski, et al. |  |  |  |  |  | 1 | 1 | 1 | 1 | 2 |  |  |  |  |  |  |  |  |  |  |  |  |  |  |  |
| Saberi, et al. | 1 | 2 | 2 | 1 | 1 |  |  |  |  |  |  |  |  |  |  |  |  |  |  |  |  |  |  |  |  |
| Evans, et al. | 1 | 1 | 1 | 1 | 2 |  |  |  |  |  |  |  |  |  |  |  |  |  |  |  |  |  |  |  |  |
| Crane, et al. | 1 | 1 | 1 | 1 | 1 |  |  |  |  |  |  |  |  |  |  |  |  |  |  |  |  |  |  |  |  |
| Gkatzidou, et al. | 1 | 1 | 1 | 1 | 2 |  |  |  |  |  |  |  |  |  |  |  |  |  |  |  |  |  |  |  |  |
| Peng, et al | 1 | 2 | 1 | 1 | 1 |  |  |  |  |  |  |  |  |  |  |  |  |  |  |  |  |  |  |  |  |
| Perski, et al. | 1 | 1 | 1 | 1 | 2 |  |  |  |  |  |  |  |  |  |  |  |  |  |  |  |  |  |  |  |  |
| Zhao, et al. | 1 | 2 | 1 | 1 | 2 |  |  |  |  |  |  |  |  |  |  |  |  |  |  |  |  |  |  |  |  |
| Fylan, et al. | 1 | 1 | 1 | 2 | 2 |  |  |  |  |  |  |  |  |  |  |  |  |  |  |  |  |  |  |  |  |
| Lyzwinski, et al. | 1 | 1 | 1 | 2 | 1 |  |  |  |  |  |  |  |  |  |  |  |  |  |  |  |  |  |  |  |  |
| Phillips, et al. |  |  |  |  |  | 1 | 1 | 1 | 1 | 2 |  |  |  |  |  |  |  |  |  |  |  |  |  |  |  |
| Herbec, et al. | 1 | 1 | 1 | 1 | 1 |  |  |  |  |  |  |  |  |  |  |  |  |  |  |  |  |  |  |  |  |
| Goldenberg, et al. | 1 | 1 | 1 | 1 | 2 |  |  |  |  |  |  |  |  |  |  |  |  |  |  |  |  |  |  |  |  |
| Hilliard, et al. |  |  |  |  |  | 1 | 2 | 1 | 1 | 2 |  |  |  |  |  |  |  |  |  |  |  |  |  |  |  |
| Milward, et al. | 1 | 2 | 1 | 1 | 1 |  |  |  |  |  |  |  |  |  |  |  |  |  |  |  |  |  |  |  |  |
| Lazard, et al. | 1 | 1 | 1 | 1 | 1 |  |  |  |  |  |  |  |  |  |  |  |  |  |  |  |  |  |  |  |  |
| Su, et al. | 1 | 1 | 1 | 2 | 1 |  |  |  |  |  |  |  |  |  |  |  |  |  |  |  |  |  |  |  |  |
| Hartzler, |  |  |  |  |  | 1 | 1 | 1 | 1 | 2 |  |  |  |  |  |  |  |  |  |  |  |  |  |  |  |
| Geuens, et al |  |  |  |  |  | 2 | 1 | 1 | 1 | 1 |  |  |  |  |  |  |  |  |  |  |  |  |  |  |  |
| Peters, et al. | 1 | 1 | 1 | 1 | 2 |  |  |  |  |  |  |  |  |  |  |  |  |  |  |  |  |  |  |  |  |
| Carolan and de Visser | 1 | 2 | 1 | 1 | 1 |  |  |  |  |  |  |  |  |  |  |  |  |  |  |  |  |  |  |  |  |
| Aji, et al. | 1 | 2 | 1 | 1 | 2 |  |  |  |  |  |  |  |  |  |  |  |  |  |  |  |  |  |  |  |  |
| Peters, et al | 1 | 1 | 2 | 1 | 1 |  |  |  |  |  |  |  |  |  |  |  |  |  |  |  |  |  |  |  |  |
| McClure, et al. |  |  |  |  |  |  |  |  |  |  |  |  |  |  |  |  |  |  |  |  | 2 | 1 | 1 | 2 | 1 |
| Wright, et al. | 1 | 1 | 1 | 1 | 2 |  |  |  |  |  |  |  |  |  |  |  |  |  |  |  |  |  |  |  |  |
